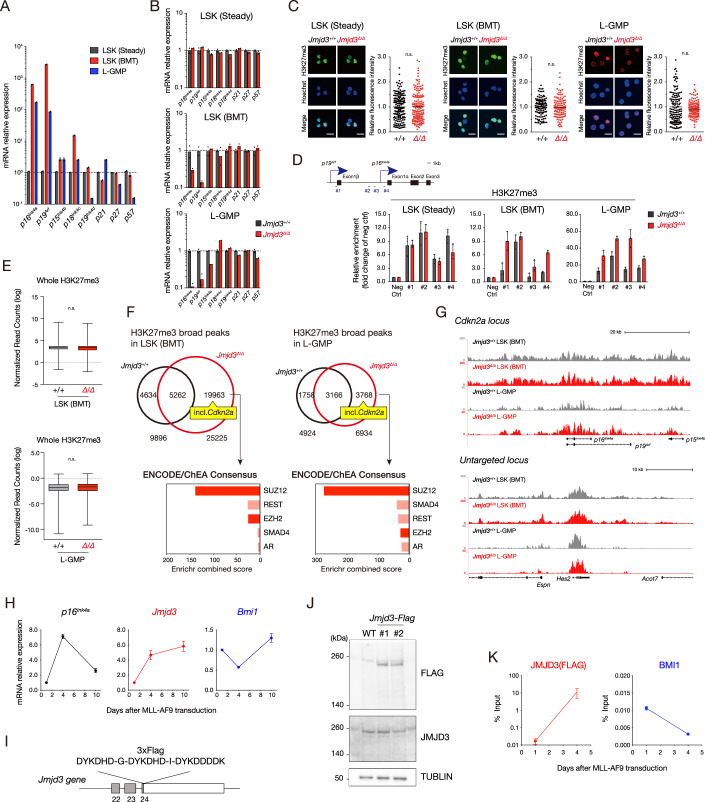# Author Correction: JMJD3-mediated senescence is required to overcome stress-induced hematopoietic defects

**DOI:** 10.1038/s44319-025-00637-9

**Published:** 2025-11-12

**Authors:** Yuichiro Nakata, Takeshi Ueda, Yasuyuki Sera, Miho Koizumi, Katsutoshi Imamura, Akinori Kanai, Ken-ichiro Ikeda, Norimasa Yamasaki, Akiko Nagamachi, Kohei Kobatake, Masataka Taguchi, Yusuke Sotomaru, Tatsuo Ichinohe, Zen-ichiro Honda, Takuro Nakamura, Ichiro Manabe, Toshio Suda, Keiyo Takubo, Osamu Kaminuma, Hiroaki Honda

**Affiliations:** 1 Department of Systems Medicine, Graduate School of Medicine, Chiba University, Chiba, Japan; 2 Department of Biochemistry, Faculty of Medicine, Kindai University, Osakasayama, Japan; 3 Field of Human Disease Models, Major in Advanced Life Sciences and Medicine, Institute of Laboratory Animals, Tokyo Women’s Medical University, Tokyo, Japan; 4 Laboratory of Systems Genomics, Department of Computational Biology and Medical Sciences, Graduate School of Frontier Sciences, The University of Tokyo, Chiba, Japan; 5 Department of Urology, Institute of Biomedical and Health Sciences, Hiroshima University, Hiroshima, Japan; 6 Department of Disease Model, Research Institute for Radiation Biology and Medicine, Hiroshima University, Hiroshima, Japan; 7 Department of Animal Experimentation, Foundation for Biomedical Research and Innovation at Kobe, Kobe city, Japan; 8 Department of Hematology, Atomic Bomb Disease Institute, Nagasaki University, Nagasaki, Japan; 9 Natural Science Center for Basic Research and Development, Hiroshima University, Hiroshima, Japan; 10 Department of Hematology and Oncology, Research Institute for Radiation Biology and Medicine, Hiroshima University, Hiroshima, Japan; 11 Department of Beauty & Wellness, Professional University of Beauty & Wellness, Yokohama, Japan; 12 Department of Experimental Pathology, Institute of Medical Science, Tokyo Medical University, Tokyo, Japan; 13 Institute of Hematology & Blood Diseases Hospital, Chinese Academy of Medical Sciences & Peking Union Medical College, Bei Jing Shi, China; 14 Department of Cell Fate Biology and Stem Cell Medicine, Tohoku University Graduate School of Medicine, Sendai, Japan

## Abstract

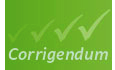

**Correction to:** *EMBO Reports* (2025) 26: 3831–3855. 10.1038/s44319-025-00502-9 | Published online 25 June 2025

The authors notified the journal of labelling errors in Figure 3 (B, C and G) that were introduced during final formatting. The figure has been corrected using the final submitted version.

**Figure 3 is withdrawn and replaced**.

**Figure 3 Published** 
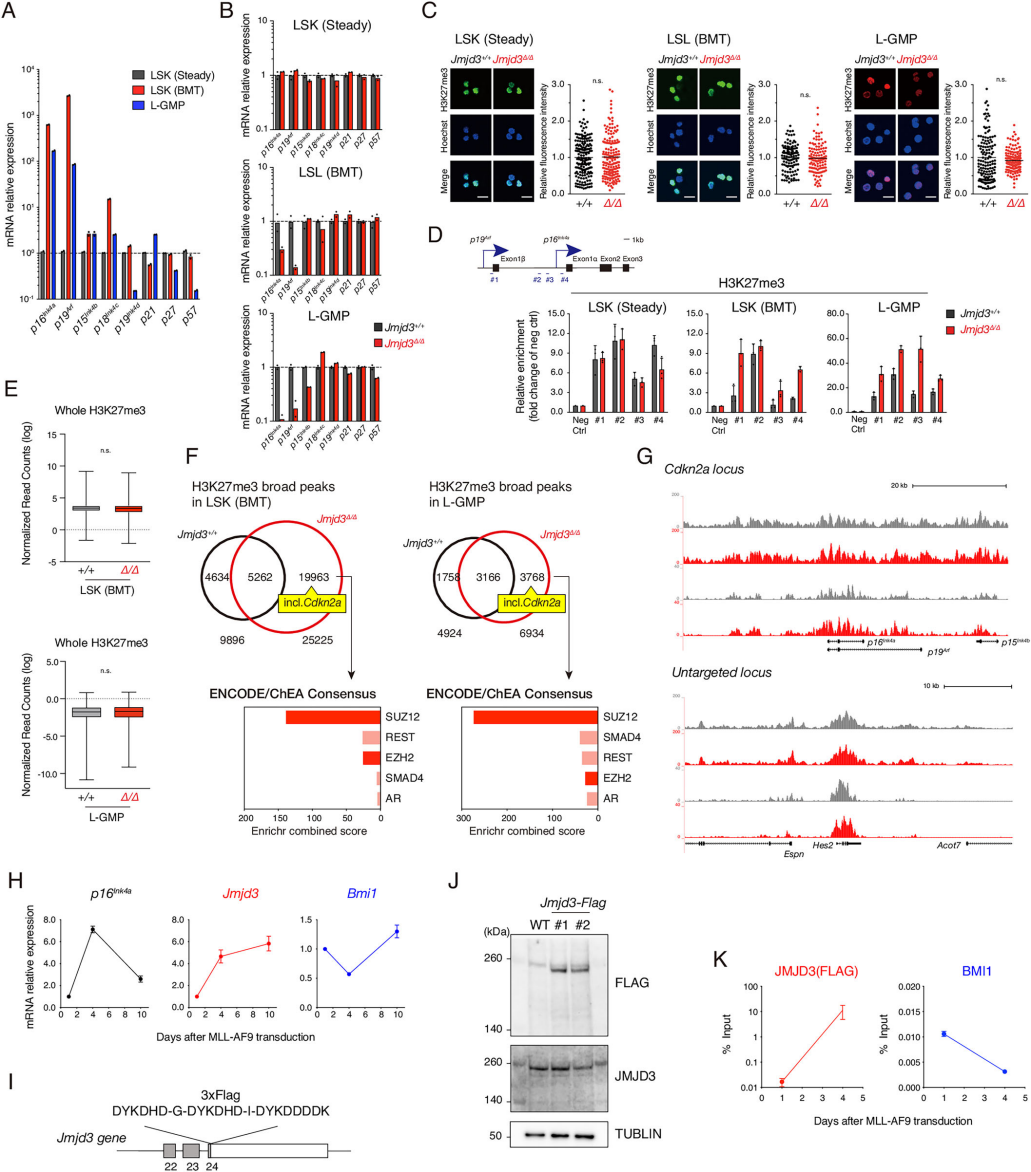


**Figure 3 Corrected**