# Supplementary material for: JMJD3-mediated senescence is required to overcome stress-induced hematopoietic defects
Source: EMBO Rep. 2025 Jun 25;26(15):3831–55. doi: 10.1038/s44319-025-00502-9 (PMC12331899; doi:10.1038/s44319-025-00502-9)
Supplement: Supplementary file 1 — Table.EV1 [file 44319_2025_502_MOESM1_ESM.pdf]

Table EV1. Surface marker phenotypes to separate mouse hematopoietic stem/progenitor cells and leukemic stem cells

Population	Surface phenotype
CMP	$CD34^+$ $CD16/32^-$ c-kit^+ Sca-1^- $Lineage^-$
GMP and L-GMP	$CD34^+$ $CD16/32^+$ c-kit^+ Sca-1^- $Lineage^-$
LT-HSC	$CD34^-$ $CD135^-$ c-kit^+ Sca-1^+ $Lineage^-$
MEP	$CD34^-$ $CD16/32^-$ c-kit^+ Sca-1^- $Lineage^-$
MPP	$CD34^+$ $CD135^+$ c-kit^+ Sca-1^+ $Lineage^-$
ST-HSC	$CD34^+$ $CD135^-$ c-kit^+ Sca-1^+ $Lineage^-$

CMP, common myeloid progenitor; GMP, granulocyte/macrophage progenitor; L-GMP, leukemic GMP; LT-HSC, long-term HSC; MEP, Megakaryocyte/erythrocyte progenitor; MPP, multipotent progenitor; ST-HSC, short-term HSC.
CD4, CD8, B220, Mac1, Gr1 and Ter119 were used to exclude lineage committed cells.
