# Supplementary material for: JMJD3-mediated senescence is required to overcome stress-induced hematopoietic defects
Source: EMBO Rep. 2025 Jun 25;26(15):3831–55. doi: 10.1038/s44319-025-00502-9 (PMC12331899; doi:10.1038/s44319-025-00502-9)
Supplement: Supplementary file 2 — Table.EV2 [file 44319_2025_502_MOESM2_ESM.pdf]

Table EV2. Gene set enrichment analysis

Common negative enriched gene sets in GSK-treated and Jmjd3$^{\Delta/\Delta}$ L-GMP cells	
	**Gene sets**
1	KEGG_NEUROACTIVE_LIGAND_RECEPTOR_INTERACTION
2	KEGG_NATURAL_KILLER_CELL_MEDIATED_CYTOTOXICITY
3	KEGG_CALCIUM_SIGNALING_PATHWAY
4	KEGG_FC_GAMMA_R_MEDIATED_PHAGOCYTOSIS
5	KEGG_LEUKOCYTE_TRANSENDOTHELIAL_MIGRATION
6	KEGG_JAK_STAT_SIGNALING_PATHWAY
7	KEGG_PHOSPHATIDYLINOSITOL_SIGNALING_SYSTEM
8	KEGG_FC_EPSILON_RI_SIGNALING_PATHWAY
9	KEGG_REGULATION_OF_ACTIN_CYTOSKELETON
10	KEGG_FOCAL_ADHESION
11	KEGG_ARRHYTHMOGENIC_RIGHT_VENTRICULAR_CARDIOMYOPATHY_ARVC
12	KEGG_VASCULAR_SMOOTH_MUSCLE_CONTRACTION
13	KEGG_LONG_TERM_POTENTIATION
14	KEGG_MELANOGENESIS
15	KEGG_AXON_GUIDANCE
16	KEGG_T_CELL_RECEPTOR_SIGNALING_PATHWAY
17	KEGG_ADHERENS_JUNCTION
18	KEGG_PANCREATIC_CANCER
19	KEGG_B_CELL_RECEPTOR_SIGNALING_PATHWAY
20	KEGG_HEMATOPOIETIC_CELL_LINEAGE
21	KEGG_LONG_TERM_DEPRESSION
22	KEGG_ECM_RECEPTOR_INTERACTION
23	KEGG_TIGHT_JUNCTION
24	KEGG_NON_SMALL_CELL_LUNG_CANCER
