# Supplementary figures and images for: JMJD3-mediated senescence is required to overcome stress-induced hematopoietic defects
Source: EMBO Rep. 2025 Jun 25;26(15):3831–55. doi: 10.1038/s44319-025-00502-9 (PMC12331899; doi:10.1038/s44319-025-00502-9)

## FLAG

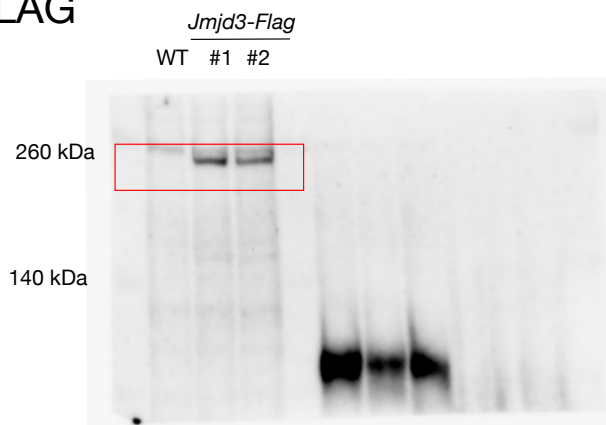

## JMJD3

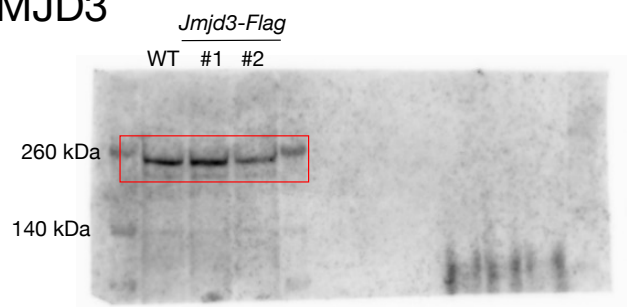

## TUBLIN

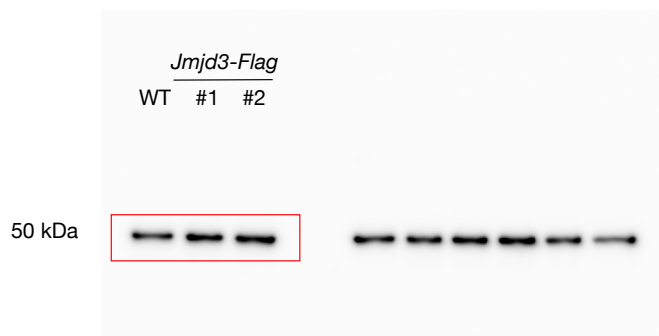

Supplement: Supplementary file 6 — Source data Fig. 3 [file 44319_2025_502_MOESM6_ESM.zip › Figure 3 Source Data/3J/3J_image.pdf]

p16<sup>INK4A</sup>

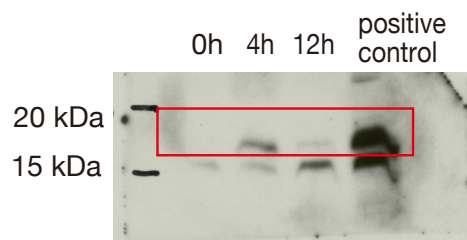

ACTIN

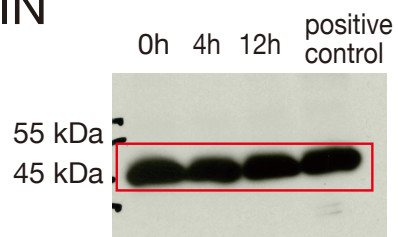

Supplement: Supplementary file 8 — Source data Fig. 6 [file 44319_2025_502_MOESM8_ESM.zip › Figure 6 Source Data/6B/6B_image.pdf]
