# Supplementary material for: JMJD3-mediated senescence is required to overcome stress-induced hematopoietic defects
Source: EMBO Rep. 2025 Jun 25;26(15):3831–55. doi: 10.1038/s44319-025-00502-9 (PMC12331899; doi:10.1038/s44319-025-00502-9)
Supplement: Supplementary file 10 — Expanded View Figures [file 44319_2025_502_MOESM10_ESM.pdf]

Expanded View Figures

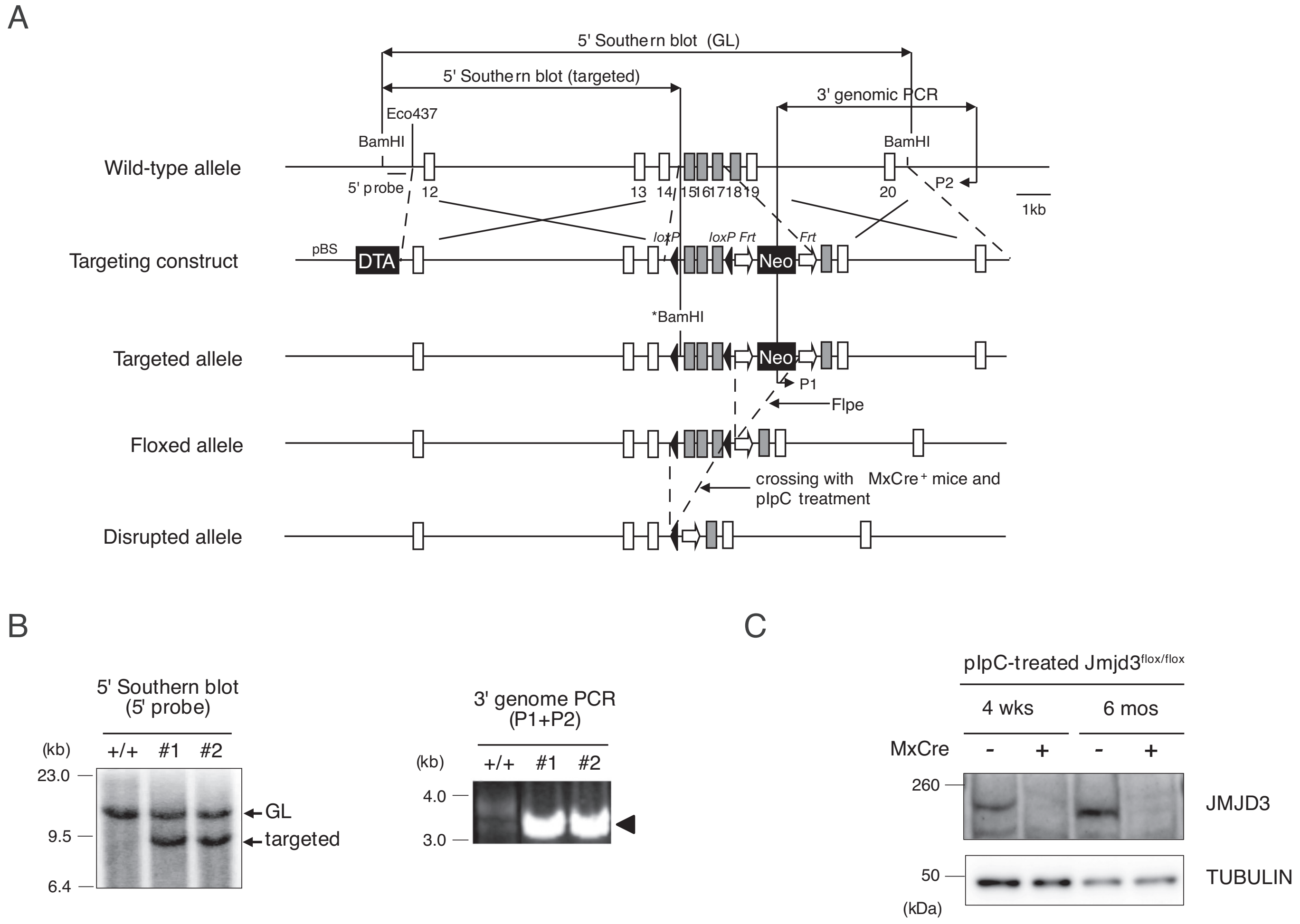

Figure EV1. Targeting strategy, genotyping of ES clones, and deletion of *Jmjd3*.

(**A**) Exons 15–17 of the mouse *Jmjd3* gene were encompassed by two *loxP* sites (black triangles), and a *neomycin*-resistance gene (*Neo*) was flanked by two *Frt* sites (white arrows). After removing *Neo* by Flpe, *floxed* exons were deleted by crossing with *MxCre*$^{+}$ mice and pIpC treatment. The position of the genomic probe for the 5′ Southern blot (5′ probe), primers for the 3′ genomic PCR (P1 and P2), and the positions of restriction enzymes (BamHI and Eco437) are shown. BamHI with an asterisk (*BamHI) is an artificial enzyme site introduced by in vitro mutagenesis. Gray boxes indicate the exons that encode the JmjC domain. (**B**) Homologously recombined ES clones (#1 and #2) identified by 5′ Southern blot and 3′ genomic PCR. Germline (GL) and targeted bands in 5′ Southern blot are indicated by arrows (left panel) and PCR products for the 3′ genomic PCR are indicated by arrowheads (right panel). (**C**) Immunoblot showing JMJD3 protein in bone marrow (BM) cells of *Jmjd3*$^{flox/flox}$; *MxCre*$^{+}$ and *Jmjd3*$^{flox/flox}$; *MxCre*$^{-}$ mice at 4 weeks (4 wks) and 6 months (6 mos) after pIpC treatment.

© The Author(s)

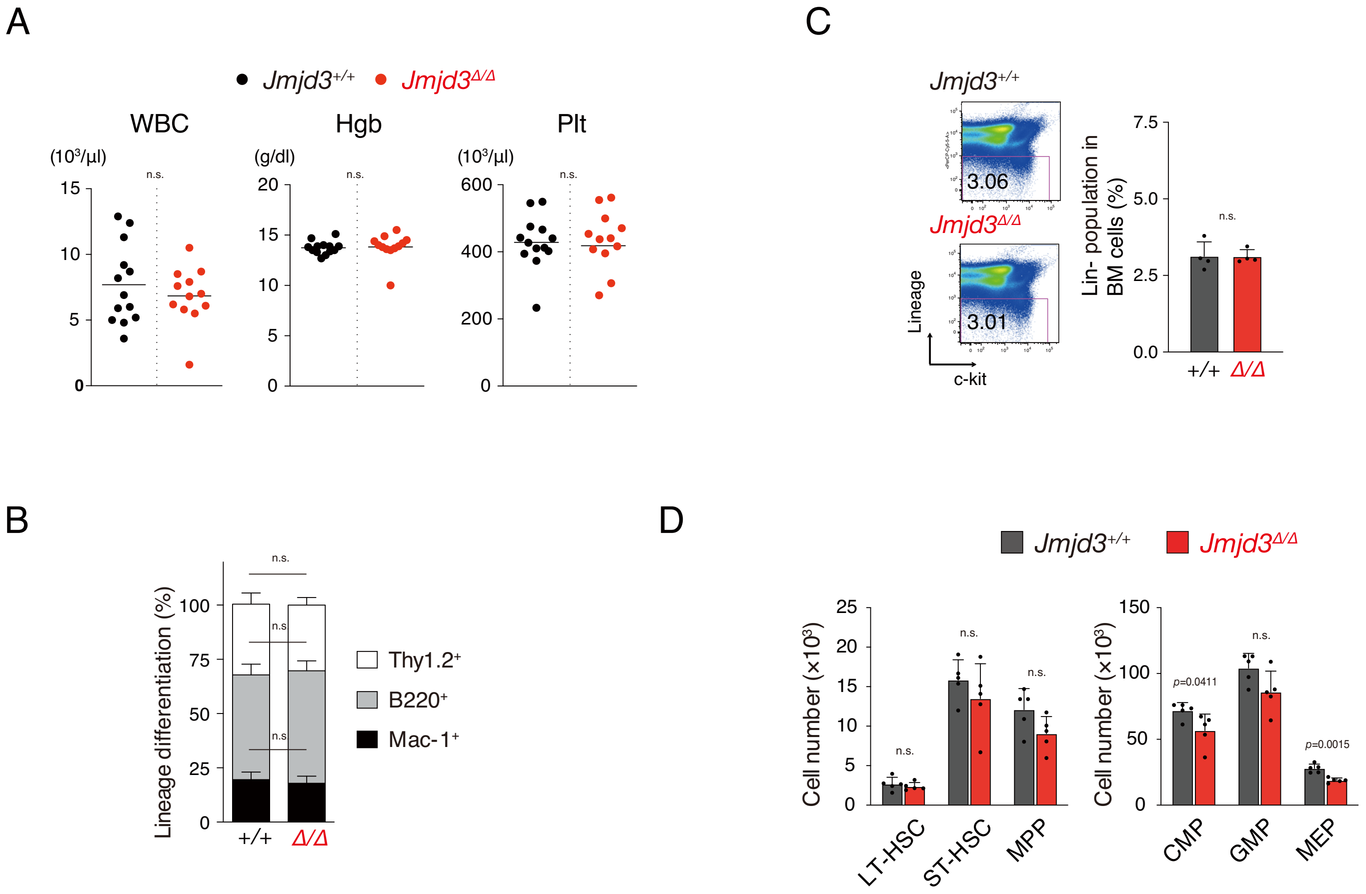

Figure EV2. Analysis of *Jmjd3*$^{\Delta/\Delta}$ hematopoietic cells at steady state.

(**A**) Analysis of peripheral blood (PB) parameters in *Jmjd3*$^{+/+}$ and *Jmjd3*$^{\Delta/\Delta}$ mice at 4 weeks after pIpC treatment. White blood cell (WBC) counts, hemoglobin concentration (Hgb), and platelet (Plt) number in the PB of *Jmjd3*$^{+/+}$ ($n = 13$) and *Jmjd3*$^{\Delta/\Delta}$ mice ($n = 12$) are plotted as dots, and the mean values are indicated as bars. Student's *t* test was used to calculate *p* value. (**B**) Analysis of lineage differentiation (Thy1.2^{+}, B220^{+}, and Mac-1^{+} cells) in the PB cells of *Jmjd3*$^{+/+}$ ($n = 13$) and *Jmjd3*$^{\Delta/\Delta}$ mice ($n = 12$) (mean ± SD). Student's *t* test was used to calculate *p* value. (**C**) Flow cytometric profiles of lineage^{-} (Lin^{-}) cells in the BM of *Jmjd3*$^{+/+}$ and *Jmjd3*$^{\Delta/\Delta}$ mice (mean + SD, $n = 5$). Student's *t* test was used to calculate *p* value. (**D**) Absolute numbers of HSPC subpopulations (LT-HSC, ST-HSC, and MPP) and myeloid progenitors (CMP, GMP, and MEP) in the BM of *Jmjd3*$^{+/+}$ and *Jmjd3*$^{\Delta/\Delta}$ mice (mean + SD, $n = 5$). Student's *t* test was used to calculate *p* values.

Gene set	LSK (Steady) (Δ/Δ vs +/+)		LSK (BMT) (Δ/Δ vs +/+)		L-GMP (Δ/Δ vs +/+)	
	NES	FDR q	NES	FDR q	NES	FDR q
PRC2_EZH2_UP.V1_UP	-0.88	0.973	-0.83	0.809	-1.16	0.218
PRC2_EED_UP.V1_UP	-1.04	0.345	-1.31	0.059	-1.61	0.003
PRC2_SUZ12_UP.V1_UP	-1.07	0.297	1.01	0.446	-1.23	0.147
PRC1_BMI_UP.V1_UP	-0.86	0.855	-1.29	0.090	-1.55	0.013
(Bracken, AP., *et al*, *Genes Dev*, 2006)						
BMI_UP.V1_UP	1.02	0.379	-1.08	0.349	-1.69	0.004
(Wiederschain, D., *et al*, *Mol Cell Biol*, 2007)						
DOUGLAS_BMI1_TARGETS_UP	1.02	0.402	-1.14	0.123	-0.91	0.702
(Douglas, D., *et al*, *Cancer Res*, 2008)						
BENPORATH_PRC2_TARGETS	-0.36	1	-1.49	0.014	-1.78	0.003
(Ben-Porath, I., *et al*, *Nat Genet*, 2008)						
KONDO_EZH2_TARGETS	0.90	0.723	-1.02	0.446	-1.45	0.027
(Kondo Y., *et al*, *Nat Genet*, 2008)						

Figure EV3. JMJD3 competitively regulates Polycomb targets under stress.

Comparison of gene set enrichment between Polycomb proteins and JMJD3. Gene sets upregulated in cells deficient in the indicated Polycomb genes were compared with those downregulated in *Jmjd3*-deficient LSK (Steady), LSK (BMT), or L-GMP. NES and FDR are indicated. Blue boxes show significantly enriched pathways (FDR < 0.25).

 © The Author(s)

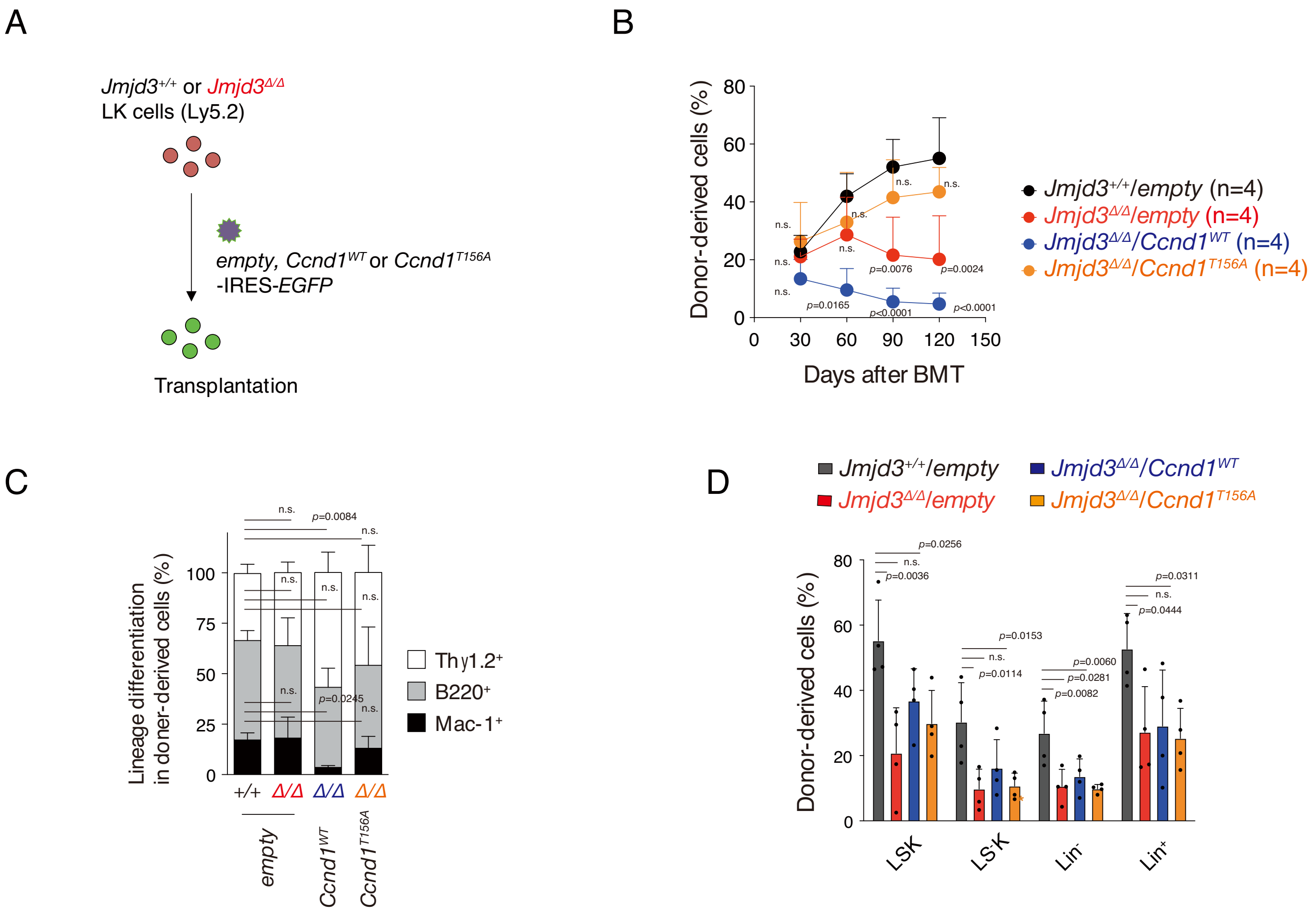

Figure EV4. HSPC defects caused by *Jmjd3* loss under replicative stress are rescued by retroviral expression of a *Ccnd1* mutant.

(**A**) A schematic diagram of *empty*, *Ccnd1*^{WT}, and *Ccnd1*^{T156A} transduction. LK cells from *Jmjd3*$^{+/+}$ and *Jmjd3*$^{Δ/Δ}$ mice were transfected with *empty*-, *Ccnd1*^{WT}-, or *Ccnd1*^{T156A}-IRES-*EGFP* retrovirus, and 5.0×10^4 EGFP$^+$ cells were transplanted into lethally irradiated recipients with 2.5×10^5 wild-type competitor MNBM cells for radioprotection (mean + SD, $n = 4$). (**B**) Chimerism of donor-derived PB cells in the recipients. Results of mice transplanted with *Jmjd3*$^{+/+}$*/empty*, *Jmjd3*$^{Δ/Δ}$*/empty*, *Jmjd3*$^{Δ/Δ}$*/Ccnd1*^{WT}, and *Jmjd3*$^{Δ/Δ}$*/Ccnd1*^{T156A} cells are shown (mean ± SD, $n = 4$). Dunnett's test was used to calculate p value. (**C**) Lineage differentiation in the donor derived PB cells in recipients 4 months after BMT (mean + SD, $n = 4$). Dunnett's test was used to calculate p value. (**D**) Chimerism of donor-derived BM cells in recipients 4 months after BMT (mean + SD, $n = 4$). Dunnett's test was used to calculate p values.

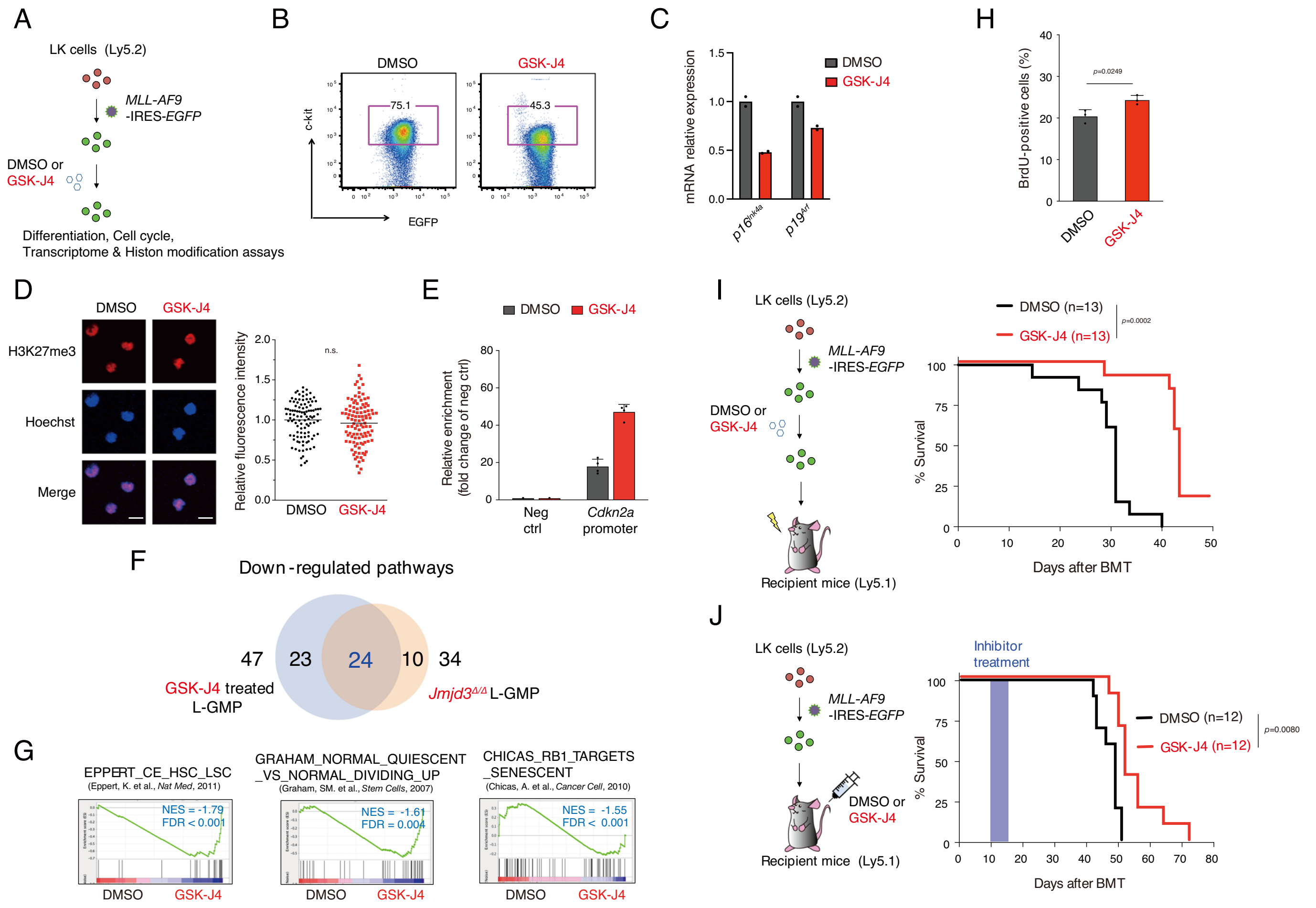

Figure EV5. Inhibition of JMJD3 suppresses LSC potential by regulating $p16^{Ink4a}$ expression.

(**A**) Schematic diagram of GSK-J4 treatment after *MLL-AF9* (*MA9*) transduction. LK cells from wild-type mice were transfected with the *MLL-AF9*-IRES-*EGFP* retrovirus. $EGFP^+$ cells were further exposed to DMSO or GSK-J4 for 24 h and subjected to the following assays. (**B**) Flow cytometric profiles of c-kit^+ fractions in *MA9* cells treated with DMSO or GSK-J4 (5 or 10 μM) for 24 h. (**C**) qPCR analysis of *Cdkn2a* genes in L-GMPs exposed to DMSO or GSK-J4 (10 μM) for 24 h. (mean + SD, $n = 3$). (**D**) Immunofluorescence staining (left panel) and relative fluorescence intensity (right panel) of H3K27me3 in L-GMPs exposed to DMSO or GSK-J4 (10 μM) for 24 h. Mean values are indicated as bars ($n = 105$). Student's *t* test was used to calculate *p* value. Scale bar, 10 μm. (**E**) H3K27me3 levels in the promoter region of *Cdkn2a* (see Fig. 3D) in L-GMPs exposed to DMSO or GSK-J4 (10 μM) for 24 h. Results are shown as fold changes relative to a negative control (Neg ctrl) (mean + SD, $n = 3$). (**F**) Venn diagrams showing the overlap of negatively enriched KEGG pathways in L-GMPs exposed to GSK-J4 (10 μM) for 24 h and $Jmjd3^{\Delta/\Delta}$ L-GMPs. The overlapped pathways are listed in Table EV2. (**G**) GSEA plots of L-GMPs exposed to DMSO or GSK-J4 (10 μM) for 24 h in the indicated gene sets (left, genes commonly upregulated in human HSC and LSC; middle, genes commonly upregulated in quiescent human $CD34^+$ hematopoietic cells; right, genes commonly upregulated through the $p16^{INK4a}$/RB1 pathway. Results are shown with NES and FDR values. (**H**) Flow cytometric analysis of BrdU incorporation in L-GMPs exposed to DMSO or GSK-J4 (5 or 10 μM) for 24 h. (mean + SD, $n = 3$). Student's *t* test was used to calculate *p* value. (**I**) Schematic diagram of in vitro GSK-J4 (10 μM) treatment for 24 h after *MLL-AF9* transduction (left panel) and Kaplan–Meier survival plots of mice transplanted with these cells (right panel). In all, 1.0×10^5 *MA9* cells ($Ly5.2^+$) were transplanted into lethally irradiated recipients with 2.5×10^5 wild-type competitor MNBM cells ($n = 13$). A log-rank test was used to calculate *p* value. (**J**) Schematic diagram of in vivo GSK-J4 treatment after *MLL-AF9* transduction (left panel) and Kaplan–Meier survival plots of mice transplanted with *MA9* cells and treated in vivo (right panel). In all, 1.0×10^5 *MA9* cells ($Ly5.2^+$) were transplanted into lethally irradiated recipients with 2.5×10^5 wild-type competitor MNBM cells. 10 days after BMT, DMSO or GSK-J4 (50 mg/kg/day) was intraperitoneally injected into the recipients for 5 consecutive days ($n = 12$). A log-rank test was used to calculate *p* values.

 © The Author(s)

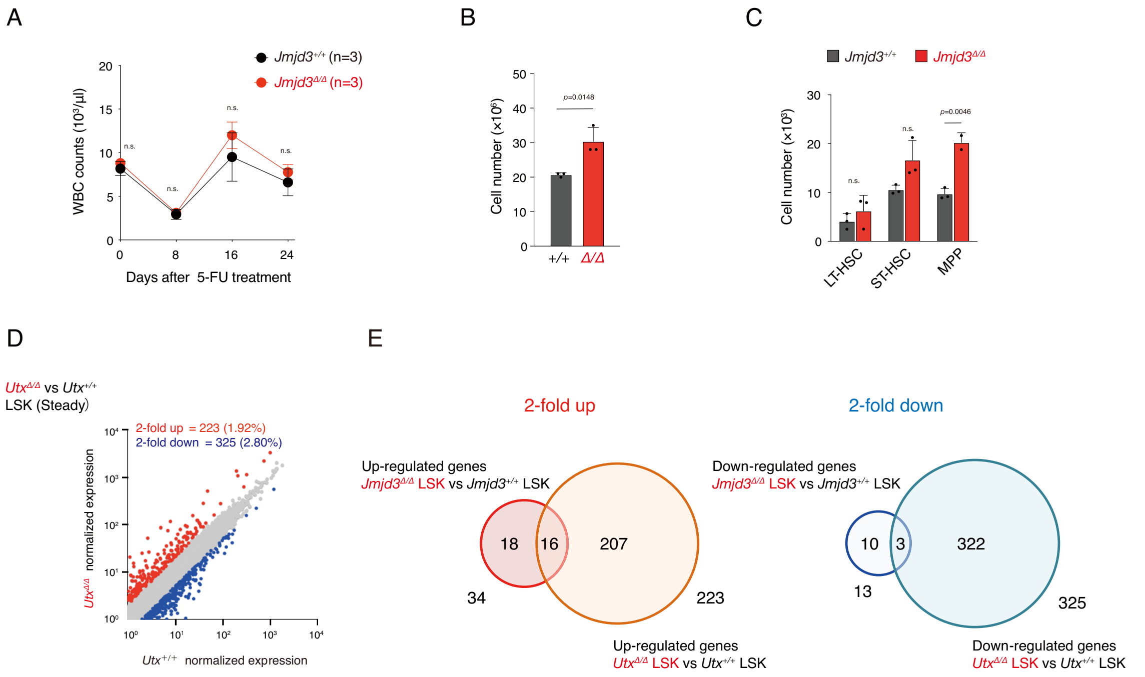

Figure EV6. Analysis of *Jmjd3*$^{\Delta/\Delta}$ HSPCs in replicative stress caused by 5-FU treatment and *Utx*$^{\Delta/\Delta}$ HSPCs at steady state.

(**A**) Changes in WBC count in *Jmjd3*$^{+/+}$ and *Jmjd3*$^{\Delta/\Delta}$ mice every 8 days after 5-FU injection (150 mg/kg) (mean ± SD, $n = 3$). Student's *t* test was used to calculate *p* value. (**B**) Absolute numbers of BM cells from *Jmjd3*$^{+/+}$ and *Jmjd3*$^{\Delta/\Delta}$ mice 24 days after 5-FU treatment (mean + SD, $n = 3$). Student's *t* test was used to calculate *p* value. (**C**) Absolute numbers of HSC subpopulations (LT-HSC, ST-HSC, and MPP) in the BM of *Jmjd3*$^{+/+}$ and *Jmjd3*$^{\Delta/\Delta}$ mice 24 days after 5-FU treatment (mean + SD, $n = 3$). Student's *t* test was used to calculate *p* value. (**D**) Scatter plots comparing normalized expression of individual genes (RPKM > 1) in LSK cells of *Utx*$^{\Delta/\Delta}$ mice compared with *Utx*$^{+/+}$ mice at steady state. Genes more than twofold upregulated and downregulated are plotted as red and blue dots, respectively. (**E**) Venn diagrams showing the overlap of genes more than twofold upregulated or downregulated in *Jmjd3*$^{\Delta/\Delta}$ and *Utx*$^{\Delta/\Delta}$ LSK cells at steady state.
